# Explaining sex: contrast between social and sexual selection

**DOI:** 10.1186/s13293-025-00807-3

**Published:** 2025-12-31

**Authors:** Joan Roughgarden

**Affiliations:** 1 Hawaii Institute of Marine Biology, University of Hawaii, Kaneohe, HI USA; 2 Department of Biology, Stanford University, Stanford, CA USA

**Keywords:** Sex, Gender, Sexual selection, Social selection, Origin of sex, Anisogamy, Hermaphroditism, Sex roles, Sex-role reversal, Female choice, Paradox of the Lek, Paradox of the creche, Bateman experiments, Parental investment, Sexual conflict, Promiscuity, Monogamy, Extra-pair copulations, Homosexuality, Mark of sappho, Gender multiplicity, Feminine males, Masculine females, Sexual dimorphism, Sexual monomorphism, Human rape, Human brain, Selfish gene

## Abstract

Darwin’s [The Descent of Man (2nd Edn.) (1871)] theory of sexual selection has been expanded into a system of interlocking hypotheses to explain many features of sexual reproduction. It focusses on mating success and relies on processes featuring bad genes, selfishness, competition, conflict, coercion, ownership and deceit. Logical flaws and an absence of factual support challenge the sexual-selection system. An alternative explanatory system, social selection, focusses on offspring delivery and relies on processes featuring negotiation, teamwork and division of labor. The sexual-selection system and the social-selection system differ in their world views of nature.

## Introduction

Most species reproduce sexually. The evolutionary study of sexual reproduction is voluminous, including research on why sexual reproduction exists to begin with, what the relation is between gametes and the male/female binary, what roles males and females play during their lives, whether male and female relations are based on conflict, the division of labor in raising offspring, the role of sexual minorities and so forth. This article offers a critical appraisal of scholarship addressing this wide range of issues. And like most of evolutionary biology, the appraisal begins with Darwin.

Darwin [42] formulated a universal biological theory of sex roles. He was concerned with traits like the peacock’s tail and the deer’s antlers, called male ornaments, which seemed to have no importance for improving survival. Instead, Darwin turned to the role such traits might have in mating.

Concerning the peacock’s tail he wrote (*cf.* pp. 216–222), “Many female progenitors of the peacock must...have...by their continued preference of the most beautiful males, rendered the peacock the most splendid of living birds.” He envisioned antlers as weapons used in male-male combat with the winner securing access to females. Although male-male combat might circumvent female choice by limiting it only to victors, females were assumed to prefer the winners because the winners were, *ipso facto*, the best of the males. Thus, female preference for victorious males caused males to become “vigorous and well-armed...just as man can improve the breed of his game-cocks by the selection of those birds which are victorious in the cock-pit”.

The peacock’s tail and the deer’s antlers were emblematic of male-female social relations generally. Darwin wrote, “Males of almost all animals have stronger passions than females,” and “the female...with the rarest of exceptions is less eager than the male...she is coy.” The phrases “almost all” and “with rarest of exceptions” show that Darwin was enunciating a theory for all of nature, excepting a few rarities one need not worry about.

Darwin’s theory of sex roles is called “sexual selection.” Its focus is on mating—how sex roles determine who gets to mate and who doesn’t. Over the years, many have expanded this theory to cover other features of sexual reproduction.

Ruben [179] coined the phrase “sex-gender system”. Ruben refers to how gender categories emerge from cultural processes that build on the “raw material” of biology. “Gender can be seen as a socially imposed division of the sexes...which exaggerates the...differences between the sexes...Male and female it creates them, and it creates them heterosexual.” Darwin’s theory of sexual selection has morphed into an extended sex-gender system that defines gender and sex roles in ways that negatively impact women and sexual minorities.

My alternative to sexual selection is “social selection”–it’s about offspring delivery–putting offspring into the next generation [165–167]. In a nutshell, social selection is about offspring delivery whereas sexual selection is about mating. Social selection is the natural selection that goes on within the social infrastructure from which offspring emerge. Proponents of sexual selection are aware that offspring ultimately need be delivered to the next generation. Yet mating remains the major focus of sexual selection owing to Darwin’s original emphasis. In social selection, mating to achieve sperm transfer is often a small component of the overall reproductive process whereas mating serves social purposes as well. Selection within the social infrastructure usually includes cooperation as much as competition and involves negotiation as much as winning. Social selection is also a sex-gender system of interconnected definitions and narratives—an alternative to the sexual-selection system.

Why bother offering an alternative to the sexual-selection system? If it ain’t broke, why fix it? First, as will be seen, the sexual-selection system is indeed broke and the normal process of science requires posing alternative hypotheses to take its place.

Second is a concern for the view nature that the sexual-selection system conveys. The title, *The Genial Gene*, Roughgarden [167] alludes to *The Selfish Gene,* Dawkins [44], a book that scientifically promotes Rand’s [153, 154] celebration of individualism and selfishness with statements like, “The world of the selfish gene is one of savage competition, ruthless exploitation, and deceit.” Sexual-selection theory brings Rand and Dawkins’ world view to animal social behavior. Social selection challenges the scientific validity of that world view. When two birds call in the morning, are they planning how to cheat and steal from each other? Or are they coordinating activities for the day’s labor? The issue is not whether the doctrine of natural selfishness is appealing or repugnant. The issue is whether it is a true, whether the birds and bees around us really are selfish and uncaring. How then would one scientifically test a world view of nature? The usual way. By testing the consequences of the premise. The totality of difficulties with sexual-selection system refutes its world view of selfishness. Whether the alternative of social selection is itself true awaits further empirical inquiry.

## Social *vs.* sexual selection

This article offers a detailed deep-dive contrast between the social and sexual selection explanations for major aspects of sexual reproduction. It presents 26 specific points that cover a broad sweep of issues starting with why sexual reproduction exists to begin with, winds through topics like sex roles and gender multiplicity, and ends with the question of why humans have brains. The article amplifies and updates topics in Roughgarden ([165–167] Chapters 2, 6, 10 and Table 19; Roughgarden and Akçay 2010ab; [168, 171, 172]). Two recent books that cover many of the issues raised in this article are by [111] and [60].

### 1. Origin of sexual reproduction

 According to the sexual-selection system, sexual reproduction evolved from asexual reproduction as a mechanism to cleanse the gene pool of deleterious mutations. The idea postulates that family lines inevitably accumulate deleterious mutations. A mating between different lines generates offspring who don’t have these mutations, rejuvenating the stock [28, 72].

According to social selection, sexual reproduction evolved from asexual reproduction to maintain a diverse gene pool in an ever fluctuating environment. The gene pool of an asexual population overcommits to particular genotypes after a run of environmental circumstances favoring those genotypes, and when the environment changes, the population suffers a crash. This lowers the asexual population’s long-term fitness below that of a corresponding sexual population^1^. The sexual population maintains a more balanced portfolio of genes than an asexual population [164]. The proximate cause of fluctuation might include the waxing and waning of parasites [77].

At an individual level, the decision to reproduce sexually is a decision to cooperate–to mix genes rather than to clone. Nowhere is this more clear than in species where reproduction is facultatively sexual. Typically, animals and plants reproduce asexually during the beginning of the growing season and switch to sexual reproduction at the end when faced with an unpredictable future. Facultative sexual reproduction, often called facultative parthenogenesis in animals and facultative apomixis in plants, occurs in a great many species including aphids [127], Daphnia [116], rotifers [186], stick insects [184], sharks [29], Komodo dragons [217], New World crocodiles [22], snakes [21], turkeys [140], the California condor [180], various plants [202], and fungi [138].

According to social selection, sex is about cooperation, not cleansing dirty gene pools. It is both a population-level and individual-level strategy to spread the risk in uncertain and fluctuating environmental conditions.

### 2. Origin of sperm/egg binary

 Some sexually reproducing species have one size of gamete, a condition called isogamy, notably in algae [65, 101] and fungi [17, 78]. However, most sexually reproducing species have two gamete sizes, one tiny–the sperm, and one large–the egg, called anisogamy.

The sexual selection system explains the origin of the egg/sperm size binary in terms of a game that the proto-sperm and proto-egg play against each other [24, 30, 118, 145]. Imagine initially that both the proto-sperm and proto-egg are the same size. Then the proto-sperm cheats, becoming a little smaller than the proto-egg, so that more copies of itself can be made. But a slightly smaller zygote now results that is less viable than the full-size zygote. Therefore, the proto-egg responds by increasing its size, restoring zygote viability to its original level. The egg’s compensating in this way is better than becoming smaller itself to match the smaller sperm, because then the zygote would suffer a deleterious double loss of resources. The sperm and egg’s “best response” to each other culminates in one gamete becoming nearly as large as the zygote, and the other gamete becoming as tiny as possible. In the sexual-selection system, the distinction between male and female arises from a battle–male and female are created as combatants in a battle of the gametes.

The social selection system theory starts with an organism who can make both eggs and sperm. That organism must allocate its gametic material to maximize the number of gametic contacts that produce viable zygotes [38, 93, 98, 155, 176, 185]. If both proto-sperm and proto-egg are the same size, only a limited number of them bump into each other in the ocean’s water where life began. Instead, the maximum number of contacts occurs when one of the gametes is nearly the zygote size while the other is as small as possible. That is, more gametic contacts occur in seawater when a cloud of large gametes is intersected by a cloud of small gametes than by two clouds of medium sized gametes intersecting each other. In social-selection theory, the gamete size dimorphism exists to maximize the number of contacts between gametes, subject to the constraint that the resulting zygotes are big enough to survive. The evolution of anisogamy is not a battle of the gametes.

### 3. Gametic packaging: male/female binary ***vs*** hermaphroditism

 Does a sperm/egg binary imply a male/female binary? Absolutely not.

The most common body plan among multicellular organisms, called “hermaphroditism”, is for an individual to make *both* sperm and eggs at some time during its life^2^. The sperm and egg may be made at the same time (simultaneous hermaphroditism) or at different times (sequential hermaphroditism). And if sequential, the eggs may be made first before switching to making sperm or *vice versa*. In either case, these individuals change sex when they switch from making one gamete type to the other. In contrast, species in which each individual makes only one size of gamete during its life are called “dioecious.”^3^ In these species, the individuals can be classified as male or female according to the gamete size produced.

A male/female binary is nowhere close to being universal across the plant and animal kingdoms. Hermaphroditism is the rule in plants and only about 6% of plant species are dioecious out of 250,000 total species [213]. The pattern is the opposite among animals [96]. Dioecy is the rule in animals, and only about 5% to 6% of animal species are hermaphroditic out of over 1.2 million total species. The number of animal species that are hermaphroditic amounts to about 65,000. However, insects, which are almost entirely terrestrial, are not hermaphroditic nor has hermaphroditism been reported in terrestrial vertebrates. Insects comprise well over half, about a million, of all animal species. This leaves a figure of about 1/3 hermaphrodite species among all animal species excluding insects.

Coral-reef fish such as wrasses, parrotfishes, groupers, damselfish, angelfish, gobies, porgies, emperors, soapfishes, dottybacks, and moray eels [215] are hermaphroditic as are many deep-sea fish [121]. Some of the hermaphroditic fish, such as the sea basses on coral reefs, are simultaneous hermaphrodites [148, 149]. More typically, hermaphroditic fish are sequential.

In the great majority of fish species, the sex-changing individuals begin as male and transition into female (MtF), such as clownfish, the colorful fish who live in sea anemones [96], cf offline appendix 2, Moyer and Nakazono [133, 134]). Only two groups, the urochordates and fish, have any species in which some individuals transition from female to male (FtM) such as the the well-studied blue-headed wrasse [160, 161, 215, 216].

Still other fish species have crisscross sex changing [137]. In one species of goby, some individuals mature from an unsexed juvenile to a female, then transition to a male, and then back to a female again (FtMtF) [105]. In another species of goby, some individuals mature from a juvenile to a male, then transition to a female, and then back to a male again (MtFtM) [136].

At the population level, evolutionary shifts from hermaphroditism to dioecy and then back again have often happened throughout evolutionary history [181] including in the Cnidarians (corals, jellyfish, hydra) and the gastropods (snails) [76, 150]. The common acorn and gooseneck barnacles found in the rocky intertidal zone are hermaphroditic and descended from shrimp-like ancestors who were dioecious. Yet these hermaphroditic barnacles in turn have been ancestral to some dioecious barnacles that are parasites upon large marine animals such as whales and turtles [81].

Across all the plants and animals combined, the number of species that are hermaphroditic is more-or-less tied with the number who are dioecious. It is not obvious which bodily arrangement to take as the starting point. Does dioecy descend from hermaphroditism or *vice versa*? Which body plan to take as the starting point is a major difference between sexual-selection and social-selection with respect to the evolution of gametic packaging.

Sexual-selection privileges the male/female binary as primitive and hermaphroditism as derived. Parker et al [145] write, “Hermaphroditism has probably evolved several times independently from the primitive situation of two separate sexes.” This stance calls for special circumstances to justify the evolution of hermaphroditism.

The special circumstance favoring hermaphroditism advocated in the sexual selection system is where “populations become sparse, when the populations are thinly scattered in temporary or marginal habitats, and when the effective breeding area is reduced by lack of motility as in sessile or sluggish animals without widely distributed gametes” [200]. In such a circumstance, “If the chance of encountering another animal is extremely small, it is highly advantageous to have that rare individual of a type that would insure fertilizing capability. In hermaphrodites this rarely-encountered individual will always have fertilizing capabilities, while the gonochoristic [separately sexed] individual’s chances are one-half (assuming an equal sex ratio). Therefore, the chances of at least one successful contact favors the hermaphrodite by a factor of at least two.”

Yes, any two hermaphrodites who mate can produce gametes that fuse, whereas separately sexed individuals must await a male-female encounter to yield gametes that fuse. But Heath [74] observed that “hermaphrodites have nothing but advantages when compared to gonochorists [separately sexed bodies].” That is, all that Tomlinson’s model shows is that hermaphroditism is absolutely better than separately sexed bodies regardless of population density even though the size of this benefit, but not its sign, does depend on population density. Therefore, the sexual-selection position requires some unspecified additional reason to account for why *all* species are not hermaphroditic.

According to social selection, hermaphroditism is the starting point, and dioecy is a specialization. This stance agrees with hermaphroditic body plans being widespread in the oldest animal phyla and classes, such as the marine invertebrates, as well as throughout the plant kingdom. Dioecy occurs primarily in more recent classes of animals such as the terrestrial arthropods and terrestrial vertebrates.

Social selection posits that packaging male and female function into separate bodies is a specialization whereby males provide a more efficient delivery of sperm to the vicinity of eggs at the cost of a lower egg production [94]. After such males arise, the remaining hermaphrodites reallocate their gonadal investment solely into eggs because producing sperm is less efficient. This scenario leads to distinct males and females from an original hermaphroditic state.

Males must supply sperm to vicinity of eggs in a higher concentration than if broadcast into the open water. Accordingly, the evolution of separate sexes from hermaphroditism is linked to a transition from broadcast spawning to localized spawning, including in the extreme, internal fertilization. Analyzing the family tree of animal phyla [73] suggests that hermaphroditism and broadcast fertilizations are both primitive and that separate sexes and localized fertilization are both derived [94] and may to some extent have evolved in parallel. Thus, in the social selection system, dioecy exists as a response to ecological conditions in which it’s efficient for one body type to transport gametes while the other body type remains relatively stationary.

Similarly, Darwin [42] notes that “it would be of no advantage and some loss of power if each sex searched for the other” (p. 230). In the same vein, the social-selection intuition for separate sexes, one of which is relatively stationary while the other is more mobile, is found in everyday life where one person is trying to meet up with another. If two people are trying to meet, it’s best for one to stand still while the other does the looking, This intuition makes sense for terrestrial animals, but not so much for marine animals, nor for plants that contract their sperm delivery to third-party pollinators. This explains why hermaphroditism for animals is primarily in marine habitats and a male/female binary in terrestrial habitats.

Next, given that a species is hermaphroditic, the social-selection and sexual-systems differ on how to explain sequential hermaphroditism. The sexual-selection system explanation for the *most common* sequence where males switch to become females (MtF) is termed the “size advantage” model. The idea is simple—animals produce one type of gamete better while small, and another when big. The minimum body size that can produce sperm is smaller than the minimum body size that can produce eggs, so the sex-change should go from male to female (MtF) as the organisms age, which is indeed true. However, crisscrossing sex changes (MtFtM and FtMtF) are not consistent with any size-advantage model unless the animals’ body size expands and shrinks back and forth across some threshold or the threshold itself moves back and forth so animals must continually change sex to keep up.

The sexual-selection account for the *least common* sequence, female switching to male (FtM) relies on male-male combat. The fish reproduce as females until they are big enough to win fights as males. This hypothesis is known as “harem polygyny”–females change sex and become males when big enough to control a harem of females [63, 64].

In contrast, the social-selection perspective is motivated by the blue-banded goby (Rogers *et al.* 2005). If two females of this species are placed together, one transitions into a male. The reason for the transition can’t be to win fights with other males–there are no other males to fight with. Instead, the sex change increases the reproductive success of the fish who switched, with the remaining female producing eggs and the newly minted male siring those eggs. Rather than sex changes to “win fights,” social-selection posits that each sex change by a female takes place when she can attain more sires as a male than she can produce eggs as a female, thereby generalizing the blue-banded goby situation. Calling the collection of females as a “harem” is a mistake—they are merely fish who contribute more to the next generation by remaining female and laying eggs rather than transitioning to become male and siring eggs.

This social-selection explanation also applies to the crisscrossing and MtF cases—the crisscrossing sex changes occur when the number of offspring left to the next generation increases by switching sex according to varying group size and composition. Similarly, the switch from male to female in MtF species occurs when the fish can lay more eggs than it can sire for a given group size. A way to calculate this relative success in siring *vs* egg laying leading to a sex switch is illustrated in Roughgarden ([167], Ch. 6 “The Body: Male, Female, and Hermaphrodite”, p. 118).

### 4. Sex roles

 Attention now focuses on species that possess a male/female binary defined according to type of gamete produced. What are the properties of the two sexes?

According to sexual selection, males and females conform to near-universal templates, which, as Darwin [42] wrote, are that males are “passionate” and females “coy.”

Today’s jargon substitutes the words “promiscuous” for “passionate” and “constrained” for “coy.” Coyne [39] confidently asserts: “We now understand...Males, who can produce many offspring with only minimal investment, spread their genes most effectively by mating promiscuously...Female reproductive output is far more constrained by the metabolic costs of producing eggs or offspring, and thus a female’s interests are served more by mate quality than by mate quantity”. Even with these shifts in terminology, the central narrative of Darwinian sexual selection remains the same—promiscuous males fight each other and display to females who choose the genetically endowed victors as their mates.

The Darwinian sex roles are nowhere close to being universal. In fish, of those species in which one or more parents care for the eggs, the male is more likely than the female to be the care provider [157]. Birds often feature bi-parental care [36, 106], whereas, in mammals, the female usually provides the care [31]. In insects, male choice is often as choosy as female choice [20]. It is hard, moreover, to distinguish “care” from “control” and the parent who is caring for the eggs or young might actually be more concerned with the control of the young than in the provision of care [165].

According to social selection, no necessary and universal sex roles exist. What each sex does is subject to negotiation in local circumstances. Any statistical regularities in sex roles that emerge reflect a statistical commonness of circumstance together with what constitutes a best bargain in such circumstances. Social behavior emerges from local ecology, and does not express a biological universal. If local ecology shows statistical regularities, so will the sex roles that emerge in those ecologies.

### 5. Mating initiation and frequency

 Sexual selection posits that males are passionate while female are coy. Nonetheless, female alpine accentors from the central Pyrénées of France solicit males for mating every 8.5 minutes during the breeding season. Approximately 93% of all solicitations are initiated by the female approaching the male, the other 7% by him approaching her, opposite to sexual selection’s expectation. Davies et al [43]

The passionate female contrasts with accounts of high mating frequency initiated and maintained by males as a form of mate guarding that prevents other males from accessing a female [103, 146].

According to social selection, universal sex roles do not exist, and depending on local conditions, either sex may be more passionate and the other coy.

### 6. Sex-role reversal

Species in which the male is drab and the female showy, the reverse of the peacock/peahen comparison, directly contradict the Darwinian norm. Many such species are known, from sea horse and pipefish to jacanas. Sexual selection claims to “explain” sex role reversal by referring to the relation between parental investment and the operational sex ratio (OSR) [34, 35, 144, 201]. The species with the higher parental investment is generally less available for mating than the sex with the lower investment, causing a net surplus of those from the low-investment sex relative to the high-investment sex. The ratio of those from one sex willing to mate to those of the other is called the operational sex ratio. In sex-role reversed species, the male happens to provide more parental investment than the female, say by carrying and/or tending the eggs, so the males are in short supply for mating relative to females. In this situation, the females may compete with one another for access to males, and become the showy sex, whereas the male remains drab. The OSR itself is not an explanation of sex role reversal, it is a re-description of the phenomenon.

The very existence of sex-role reversed species *contradicts* the basic tenet of sexual selection that sex roles can be traced to gamete size, because pipefish and jacana males, like all other males by definition, produce tiny sperm. Gamete size does not dictate sex role. This contradiction is a fatal flaw in sexual selection theory.

According to social selection theory, reversed sex roles are not problematic because sex roles reflect and are negotiated in local ecological situations. For example, male katydids show intra-specific variation in sex role [70]. Male parental investment consists of nutritious spermatophores that males provide to females. When other food is scarce, females compete for males to obtain the spermatophores. However, an increase in local food from a low level to a high level results in a switch from sex-reversed roles to the sex-typical roles. Thus, sex roles are determined by local ecological situations, in this case the level of food available to females relative to the nutritional value of male spermatophores.

### 7. Female choice

 Darwin [42] claimed that the peahen’s aesthetic caused peacocks to evolve the most beautiful of tails. Today’s sexual-selection narrative avoids the term “aesthetic” but instead envisions that females select mates for their genetic quality. Whatever sense of “aesthetic” a female bird might have is now understood to be a finely honed ability to distinguish which males have the best genes. Mating rituals are interpreted as ways by which a male can advertise his good genes. What counts as the best genes?—(1) genes that confer health and vigor to the offspring, (2) genes that confer traits she finds “sexy” to ensure that her sons are also sexy (called the “sexy son hypothesis”, [218], and/or (3) genes that are compatible with her own genes, say with respect to the immune system [147].

However, a review of 95 studies covering 50 species of birds finds limited to no support on behalf of female choice for any genetic benefits of any type. [2]. Others, too, find little support, either theoretically or empirically, for indirect genetic benefits in comparison to direct benefits [102].

In contrast, according to social selection, females choose mates based on maximizing the number of young she can produce that are successfully reared by her own efforts, in conjunction with help from the mate, plus assistance from the social infrastructure. According to social selection, female choice is indifferent to male genes, but is focused on whether the male is likely to deliver on any promised direct benefits [59]. She should use an expectation of direct benefits from a male discounted by the probability that the male will renege on, or somehow be prevented from, delivering on his promise, as the criterion for choice. A premium will be placed on the compatibility and health of the prospective partner. Health is important not as an indicator of “good genes,” but as a sign of competency to provide direct benefits. According to social selection, both males and females must choose each other, because the offspring represent a common investment in which both have a shared interest.

### 8. The classic chestnuts

 Surely, one might think, any extended critique of sexual selection must be overlooking a wealth of evidence in its favor. Indeed, a wealth of studies do claim to support sexual selection, but drilling down to the data reveals uncertainty and doubt.

Take the peacock/peahen case. Takahashi et al [142] “found no evidence that peahens expressed any preference for peacocks with more elaborate trains... similar to other studies of galliforms showing that females disregard male plumage....our findings indicate that the peacock’s train (1) is not the universal target of female choice, (2) shows small variance among males across populations and (3) based on current physiological knowledge, does not appear to reliably reflect the male condition.” Instead, the study proposes that “there has been selection on females for dull-coloured plumage, as would be expected in ground-nesting species with little or no male parental care where females suffer high predation risk during incubation” Thus, researchers on peacocks no longer consider the male’s tail to be a derived specialization to attract females, but instead regard the female’s lack of a tail to be a derived specialization to avoid predators.

In addition to presenting their own data, Takahashi et al [142] review many studies with peacocks, and report that with those in the United Kingdom generally support the sexual-selection narrative, while those elsewhere do not. They comment that “positive results are likely to be published and distributed in the research field of sexual selection” and caution that “it is equally important to publish negative results,” thus raising suspicion of a publication bias favoring sexual selection in behavioral-ecology research.

Consider next some less-well-known examples. The collared flycatcher is a small migratory woodland bird of central and eastern Europe studied for over 24 years on the Swedish island of Gotland, yielding a cumulative sample of over 8,500 individually marked individuals [151]. The investigators write, “Sons inherit their fathers’ forehead patch size. Females prefer to mate with males with a large forehead patch, especially late in the season”. However, the investigators also found a near absence of heritability in male fitness. That is, although males may vary among themselves in how many offspring they sire, almost none of this variation can be inherited, rendering it pointless for a female to attempt to ascertain which males have the best genes. Not surprisingly then, there is almost no heritability for female choice of male badge size. That is, if a female does happen to prefer males with large badges, this preference is not inherited among her daughters. Thus, preference by females for the badge does not endow their sons with genes that will cause them to be desired as mates in the future, contrary to the sexy-son theory.

The blue tit is a woodland bird in the United Kingdom [71]. The blue caps on heads of males and females appear to be the same when viewed in regular visible light, but differ greatly when seen in ultraviolet light. The investigators write, “females can evaluate the fitness of a male’s offspring according to the reflectance properties of his crown.” Contrary to the prediction of sexual-selection theory, the authors found that “variation in coloration is only weakly heritable” and that two components of offspring fitness–nestling size and fledgling recruitment are strongly dependent on parental effects, rather than genetic effects. The authors conclude, “Models of indirect sexual selection predict that sexually selected traits should have high heritability, that the magnitude of genetic variance in fitness should be substantial, and that there is significant positive genetic covariation between the sexually selected trait and fitness. In contrast to these predictions, this study has demonstrated that chromatic variation in the cap and chest of the blue tit is only weakly heritable, that variation in chick recruitment is determined to a large degree by environmental, rather than genetic effects, and that the genetic covariation between colour and fitness components is either non-significant or negative. Taken together, these results suggest that neither cap colour nor chest colour are likely to accurately reflect any genetic benefits a female may gain by mating to highly ornamented males.”

The barn swallow has a characteristic V-shaped tail and two trailing streamers. On average, the tails of males are longer than those of females. The tail length in females is taken to be aerodynamically optimal, and the elongated tail in males has been hypothesized to be exaggerated because of sexual selection–the longer tail is supposed to indicate an enhanced capability that comes from possessing good genes, called the “handicap principle.” A handicap is supposed to reveal genes so good that a male can sustain the handicap and still function well. If so, females can choose males with the longer tails and thereby gain superior genes for their offspring. However, a study of the barn swallows [23] established that “contrary to handicap models of sexual selection, the sexually selected exaggeration of this trait [tail length in males] provides females with little information about any aspect of mate quality.”

The lark bunting is a migratory songbird that breeds on prairies of the Great Plains of North America. Females are brown with dull white wing patches. Males are more colorful–generally black or black with patches of brown feathers, and have conspicuous white wing patches that vary in size and whiteness. A five-year study of lark buntings in the short-grass prairie of Colorado [27] found that the traits correlated with reproductive success varied across years, and that female choice did as well. The authors argue that female choice tends to align more with traits that predict maximizing the number of eggs she will fledge rather than with traits that predict whether the male is a successful sire, agreeing with studies of sand gobies [59]. Chaine and Lyon [27] conclude that the “theory on the evolution of ornamental male traits by sexual selection assumes consistency in selection over time.” However, “in lark buntings sexual selection on male traits varied dramatically across years and, in some cases, exhibited reversals in the direction of selection for a single trait.”

The lark bunting is not alone. Two other studies appeared later in 2008 describing fluctuating directions of selection: a wild population of Soay sheep on the island archipelago of St. Kilda, NW Scotland [162] and a population of red jungle fowl at a field station of the University of Stockholm [37]

Finally, take the famous Bateman principle. Bateman [14] presented an experiment with *Drosophila melanogaster* regarded as confirming the theory of sexual selection. Bateman reported that for a male, “fertility is seldom likely to be limited by sperm production but rather by the number of inseminations or the number of females available to him.” He also claimed to find an “undiscriminating eagerness in males and discriminating passivity in females”. Bateman further reported that male fitness (number of eggs bearing the male’s paternity) increased with the number of mates, whereas for females, fitness was independent of the number of mates beyond one–one male was sufficient to supply all the sperm needed to fertilize the eggs. Bateman’s results are often referred to as Bateman’s principle.

But as Tang-Martinez and Ryder [196] pointed out, “Bateman had no way of knowing how many times a female mated, or with how many males, because he did not conduct behavioral observations.” Similarly, Dewsbury, [50] points out that “Bateman apparently made only casual observations of the actual behavior of his flies.” Then still another critique of Bateman’s work [193] noted that “his methods had flaws, including the elimination of genetic variance, sampling biases, miscalculations of fitness variances, statistical pseudo-replication, and selective presentation of data. We conclude that Bateman’s results are unreliable, his conclusions are questionable, and his observed variances are similar to those expected under random mating.”

Tang-Martinez and Ryder [196] continue, “Among Bateman’s most important popularizers is Trivers [201] whose influential paper on parental investment perpetuated the stereotypes of indiscriminate males and sexually restrained females”. They decry the “dogmatization” of Bateman’s findings in textbooks (*eg.* [6]).

Despite the enthusiasm Bateman’s [14] paper attracted, the paper is increasingly discounted [84, 86], although a descriptive statistic he introduced is sometimes used. The “Bateman gradient” is the regression of reproductive success against number of mates. If the gradient is steep, each additional mate produces a large increase in the reproductive yield. And if the gradient is different between the sexes then increasing the number of mates matters more to one sex than the other.

A paper ambitiously titled, “Darwinian sex roles confirmed across the animal kingdom” compared the Bateman gradients for males and females in 66 species [95]. The authors conclude “Our findings provide the first comprehensive evidence that Darwin’s concept of conventional sex roles is accurate and refute recent criticism of sexual selection theory.” (The allusion is to criticism of sexual selection in Roughgarden [165, 167] and elsewhere.) The authors found that most species had higher Bateman gradients in males than females. But not all. Pipefish and seahorses, which are sex-role reversed, have higher gradients in females than males. Drilling into the article’s supplementary material reveals that the bony fish as a group show no difference in the male and female gradients, consistent with the fact that in fish males provide the parental care, if any. The group of 66 species does not include monogamous birds with little sexual dimorphism such as sea birds like albatrosses or penguins. One could assemble an alternative set of 66 species with different results. Moreover, the behavior of the 66 species is not reported, so no one knows whether the behavior actually consists of undiscriminating eager males and discriminating passive females. What the study shows is simply that more species have males with a higher Batman gradient than females, which is already known because non-sex-role reversed species species are more common than sex-role reversed species. In short, the authors overgeneralize their own data, do not explain the exceptions, and come nowhere close to confirming Darwinian sex roles across the animal kingdom.

Social selection treats the Bateman principle as having no generality, and treats the Bateman gradient solely as a descriptive statistic for quantifying the relation of mating to reproduction in some situations.

In short, the evidentiary support is weak to nonexistent for sexual selection’s central tenet that female choice for good genes drives the evolution of expensive ornaments. And in lark buntings the data wind up supporting the social selection premise that female choice is for offspring yield, not the sire’s genes. The weak evidentiary support for sexual selection strikingly contrasts with the strong evidentiary support over 70 years for natural selection exemplified by famous studies on industrial melanism in Lepidoptera by Kettlewell [100] and thousands of other studies since then.

Beyond the classic chestnuts, social and sexual selection differ on how to explain many other aspects of sexual reproduction, and so the comparison continues...

### 9. Modeling social behavior

 Sexual selection models social behavior using competitive evolutionary game theory [192]. Particular behaviors are strategies. The prisoner’s dilemma game is an oft-cited example in which the strategies of play are to cooperate or to defect. The “payoff matrix” tabulates the payoff to each player for all combinations of these strategies. The solution to the game is an evolutionary stable strategy (ESS), which is a combination of strategies for both players such that a mutant allele for some other combination cannot increase when rare. This approach is a single-tier approach in that behaviors are evolutionary strategies. This requires thinking of particular behaviors as having a direct genetic basis, such as the “gene for” cooperating, for defecting, for shyness, for aggressiveness, etc. Behaviors rarely have a direct genetic basis. The single-tier approach forces narratives of genetic determinism.

Social selection models social behavior in two coupled tiers, behavioral and evolutionary [168, 173]. Dynamics in the behavioral tier of social-selection theory employs both cooperative and competitive game theory. The solution concepts include the Nash bargaining solution (NBS) as well as the more familiar Nash competitive equilibrium (NCE) (Roughgarden [169]). In cooperative game theory, cooperative solutions may be attained by the parties playing with coordinated tactics and with the perception of shared goals made possible through animal friendships. These friendships are facilitated with physical intimacy involving the mutual exchange of pleasure, like reciprocal grooming, preening, same-sex sexuality, and interlocking calls and other vocalizations [170]. Other possibilities include noncooperative dynamics that wind up at a purely competitive outcome.

Below the behavioral tier, social selection uses an evolutionary tier in which payoff matrices themselves evolve together with the rules of play. The social behaviors then evolve indirectly. As the payoff matrix and rules of play evolve, particular behaviors unfold [5].

### 10. Purpose of reproductive social behavior

 According to sexual-selection theory, reproductive social behavior comprises a “mating system” [55]. Within a mating system, natural selection arises from differences in “mating success”. Particular behaviors are understood by how they contribute to attaining mating success. In a mating system, the females are regarded as a “limiting resource” for males, and males compete for access and control of mating opportunities. Sexual selection elevates one component of reproduction, namely mating, into an end in itself.

According to social selection theory, reproductive social behavior comprises an “offspring-rearing system.” Within an offspring-rearing system, natural selection arises from differences in number of offspring successfully reared. The principal male-female social dynamic is to negotiate bargains and side payments to arrive at a division of labor to rear the offspring.

### 11. Male genetic quality

According to sexual-selection theory, males can be ranked in a hierarchy of genetic quality. Females seek males with good genes and avoid those with bad genes. The problem with this story is that if, generation after generation, female choice weeds out the males with bad genes, then eventually no bad genes remain. When all males have become genetically equivalent, females no longer need to bother selecting males on the basis of their genes. This dilemma *contradicts* sexual selection theory and is called the “Paradox of the Lek”.

Therefore, sexual-selection theorists have concocted genetic schemes, typically involving high mutation rates spanning polygenic loci to replenish the supply of bad genes that are supposedly being continually eliminated by female choice [126, 178, 199]. Table 1 of Miller and Moore [126] lists nearly a dozen other papers attempting to resolve the paradox.

According to social selection, the fundamental contradiction that the Paradox of the Lek poses cannot be resolved and no male hierarchy of genetic quality exists. All males are equivalent in genetic quality, excepting for a very rare fraction that contains obviously deleterious mutations and are present in a rare mutation-selection balance (say, 1 in $10^6$).

The lek paradox is only one of the population-genetic difficulties with sexual selection. The lek paradox assumes that bad genes in males once existed but are eliminated through female choice, requiring a process to replenish those genes that were just eliminated. A more fundamental difficulty challenges the plausibility of whether bad genes ever existed to begin with that could be detected by female choice.

### 12. Limited scope for fitness-based choice

 Female pronghorn antelopes select the males who will sire their young [26]. The investigators write that “in the 2 weeks preceding estrus, each female visits several potential mates that hold widely spaced harems...This female sampling behavior is energetically expensive.” The investigators report that females favor “running speed, endurance, agility, and tactical spatial sense.” As a result, females “converge on a small proportion of males that sire most young.” The investigators conclude: “Preferred males, those that have demonstrated impressive vigor under the relentless scrutiny of the female mate sampling process, likely are those with relatively low numbers of small-effect deleterious mutations.” Therefore, “female choice may be important and unrecognized as a force that can lower population genetic load.”

Female choice is thus represented as a natural eugenics—cleansing the gene pool of small-effect deleterious mutations that have accumulated, reducing the “genetic load” that these mutations place upon the species [135]. This focus on the gene pool’s bad genes, in this instance on weakly deleterious mutations can be traced to the founder of eugenics, Francis Galton (1865). During the 1950 s, studies claimed that every person has three to five recessive lethal genes that would cause their children to die if they chose the wrong marriage partner [132], an idea extended to the role of female choice in sexual selection [152].

The gene pool does accumulate small-effect deleterious mutations, but can female choice cleanse it? Is there enough difference between the fitness of genetically good males and bad males for a female to discern? If not, then female choice cannot impact on the gene pool’s quantity of deleterious mutations.

Small-effect deleterious genes reside in the gene pool in a mutation-selection balance—they are being replenished through mutation and eliminated by natural selection, leading to a steady state distribution. The steady state distribution of the number of deleterious genes carried by individuals in a population is a Poisson distribution [72]. The location of the peak of this distribution indicates the average number of deleterious genes in an individual. The peak’s location has been derived as $(L \, \mu / s)$ where *L* is the number of genetic loci in the genome, $\mu$ is the probability that a deleterious mutation arises in an individual at any particular locus per generation, and *s* is the amount by which the fitness of an individual carrying a deleterious gene is reduced relative to an individual without a deleterious gene. That is, if 1 is the fitness of an individual without a deleterious gene, then $(1 - s)$ is the fitness of an individual with one deleterious gene. If an individual has two deleterious genes, then its fitness is $(1 - s) (1 - s)$, indicating a double dose of deleteriousness. Similarly, if an individual as *k* deleterious genes, its fitness is $(1 - s)^k$.

This formula allows one to calculate the average number of weakly deleterious genes in an individual using typical values for *L*, $\mu$, and *s*. Let *L* be 25,000 loci, $\mu$ be $10^{-6}$, and *s* be 0.001. Then the average number of weakly deleterious genes in an individual works out to be 25—typically a different 25 genes for each individual, but still 25 on the average. Now, what can female choice do about this?

Well, if the genetically average male has 25 weakly deleterious genes, how few does a genetically good male have, and how many does a genetically poor male have? To see the spread in male genetic quality, consider the variance of the distribution which, being a Poisson, has the property that the mean equals the variance. So the variance in our example is also 25. The standard deviation then is 5 (i.e., $5 = \sqrt{25}$). So, a genetically good male has 20 deleterious genes, and a genetically bad male has 30 (i.e., the mean ± one standard deviation).

Next, what is the fitness of a genetically-good male compared to a genetically-bad male? Well, $(1 - s)^{20}$ for a genetically-good male works out to be 0.98 and $(1 - s)^{30}$ for a genetically-bad male is 0.97. These are nearly the same, only a 1% difference. There’s simply no way a female in the field can perceptually discern a one percent difference in the fitness between genetically good and bad males. Moreover, the sample size of males that a female actually encounters is small, limiting her exposure to whatever genetic variety does exist.

Therefore, the claim that female choice in pronghorn antelopes is a force lowering a population’s genetic load is incorrect. The benefits that accrue to females through their choice of mating partners has nothing to do with the male’s genes but has everything to do with how well the male was raised–his diet, exposure to natural hazards and parasites, all of which are environmental, not genetic factors.

The social selection position views the entire discussion of female choice as cleansing the gene pool of weakly deleterious mutations as a waste of time.

### 13. Parental investment

According to sexual selection, a female has a higher parental investment than a male because the egg is bigger than the sperm. The sperm are considered cheap and the egg expensive. This initial difference is then extrapolated to an entire suite of female and male behaviors [39].

According to social selection, male and female parental investments are more or less the same initially. An ejaculate might typically contain $10^6$ sperm, whereas an egg is typically $10^6$ times as large as a sperm. So the size of the ejaculate and egg are often about the same order of magnitude [49]. Therefore, according to social selection, the male and female begin with more or less equal investments in the offspring. Hence, sex roles emerge in local context, not as a matter of logical necessity tracing to gamete size.

Darwin [42] also opined that male and female expend about the same reproductive effort. He wrote “The female has to expend much organic matter in the formation of her ova, whereas the male expends much force in fierce contests with his rivals, in wandering about in search of the female, in exerting his voice, pouring out odoriferous secretions etc.” (p. 232).

### 14. Sexual conflict and cooperation

According to sexual selection, male and female are fundamentally in conflict because gametic dimorphism originates as a conflict between egg and sperm. Sexual selection takes male-female conflict as the baseline condition sees male-female cooperation as a possible (and unlikely) secondary development.

Data do not confirm ubiquitous male-female conflict in nature. In avian bi-parental care, sexual selection predicts that each bird is trying to get the other to do most of the work. If one bird happens to “generously” feed the nestlings with an extra worm, the other is supposed to reduce the number of worms it supplies and use the saved energy to seek extra matings somewhere else. Experiments show however, that the birds tending a common nest often try to help each other, and do not take advantage of each other [80, 129, 183, 225]. A more recent critique of the preoccupation with sexual conflict concludes that “it will take many years to redress the bias toward sexual conflict that has taken the major share of empirical attention to this point” (Griffith 2017).

According to social selection, male and female mates fundamentally begin with a cooperative relationship because they have committed themselves to a shared account of evolutionary success. Their offspring represent a common investment whose evolutionary earnings cannot be subdivided. Hurting the other hurts oneself, and helping the other helps oneself, in terms of number of offspring successfully reared. As such, conflict develops only secondarily if a division of labor cannot be successfully negotiated [4].

### 15. Male promiscuity

 According to sexual selection, males are universally promiscuous because sperm are cheap and eggs expensive.

From a social-selection perspective it makes no sense for a male to abandon control of his reproductive destiny. According to social selection, male promiscuity is a strategy of last resort when males are excluded from sharing control of offspring rearing. Thereupon, the only strategy remaining is to fertilize as many females as possible.

### 16. Monogamy

 In sexual-selection theory, monogamy violates the dictate that males should be promiscuous. Therefore, sexual selection explains away monogamous pair bonds as entrapment of males by females or as a default when no other mates are available or attainable [31, 55, 141].

Social selection distinguishes two forms of monogamy: economic monogamy, which represents an agreement to rear offspring in teams of one male and one female, and genetic monogamy, which represents an agreement not to mate outside the pair bond [165]. Most monogamy is economic monogamy and nothing requires economic monogamy and genetic monogamy to coincide. In social selection, monogamy emerges in ecological situations where the work of offspring rearing is most efficiently done in male-female teams, rather than as solitary individuals or in teams of more than two individuals.

### 17. Extra-pair parentage

 Extra-pair paternity (EPP) occurs when a male sires young in a nest other than the one where he is working with a female, and extra-pair maternity (EPM) occurs when a female deposits eggs at a nest other than the one where she is working with a male. Both EPPs and EPMs result in extra-pair parentage. An extra-pair copulation (EPC) may or may not yield a fertilization.

According to sexual selection, extra-pair parentage is “cheating” on the pair bond, the male is said to be “cuckolded,” offspring of extra-pair parentage are said to be “illegitimate,” and females who do not participate in extra-pair copulations are said to be “faithful” [56, 113, 214]. This judgmental terminology fails to distinguish economic from genetic monogamy, and applies a contemporary illusion of human marriage to animals. EPPs are assumed to reflect the basic male promiscuity, whereas EPMs are described as “sexual parasitism.” The females who deposit eggs in a neighbor’s nest are called “brood parasites.”

Continuing, sexual-selection theorists postulate that females invite EPCs when paired with a genetically inferior male. Qvarnström et al [151] claim that for collared-flycatchers “extra-pair offspring sired by highly ornamented males fledge in relatively better condition and therefore have higher survival chances than within-pair offspring.”

A meta-analysis of over 100 studies of extra-pair paternity in birds spanning over 50 species. [2] finds that only 40% of the studies report some kind of indirect genetic benefit (either good genes or compatible genes) accruing to females who pursue extra-pair matings, and the other 60% found that genetic benefits of any type were absent. The meta-analysis showed that extra-pair males are on average no different than within-pair males in ornaments and that extra-pair offspring don’t survive better than within-pair offspring. Thus, statistics on extra-pair paternity studies do not support the theory that females solicit EPCs to obtain a genetic upgrade over their nest-partner.

Griffith [68] noted the discrepancy between EPC and fertilization, writing “More than 10 years ago, Dunn and Lifjeld [54] demonstrated convincingly that there was not a linear relationship between the observed rate of EPCs and EPP across a number of species, and to this date, there remains little or no evidence to support a simple relationship between the two...to date, in the only species in which we can make an estimate about the relationship between extra-pair behavior and actual EPP, it seems that about 80% of females may be having EPCs and yet only about 30% of females normally have EPP in their broods [123]...Therefore, when we detect extra-pair offspring in the broods of a sample of females in a population (e.g., 30%), we are unable to conclude that these are the only 30% of females in the population that have had EPCs. In fact, it is quite possible that all females may have had EPCs and that in only 30% of them did the sperm of extra-pair sires achieve fertilizations.”

The disconnect between mating and fertilization raises the question of what the biological function of most mating is—primarily to bring about fertilizations, to achieve social outcomes, or some mixture of both? In the European oystercatcher one of the breeding arrangements consists of two competing females in separate nests with the male splitting time between them, and another breeding arrangement consists of two cooperating females in one nest with a male (Hegand Treuren 1998). The competing females don’t mate with each other, whereas the cooperating females do frequently copulate with each other. Thus, the same-sex copulations that can’t lead to fertilizations do contribute to the social infrastructure within which their offspring are raised.

In social selection, the bird nests in a woods comprises a system, a “breeding neighborhood” [165]. The nests might be physically touching one another to comprise a colony. But the distance between nests could be expanded, as an exploded colony, so to speak, depending on how resources are distributed. Birds should distribute their eggs into several nests to spread the risk of predation and damage from storms, fire and so forth as a social insurance policy. Furthermore, by distributing eggs among nests, the adult birds in the area acquire a collective interest in patrolling for predators, and also shared parentage removes an incentive to attack each other’s nests. The idea of a breeding neighborhood refers to the spatial scale over which parentage of nests is shared analogous to the concept of a genetic neighborhood in population genetics [117].

To explain extra-pair parentage, social selection borrows from the economic theory of marriage [15, 16] and restates the theory of reproductive transactions [187, 212] in terms of offspring numbers rather than genes while also adding the possibility of trading eggs and fertilizations as side payments.

Becker introduced the “marriage market” as a matrix of payoffs to each party in all possible pairing arrangements. Social selection views the payoff to a pair as the number of young fledged and the payoff matrix as the yield in fledged young from for all possible pairing arrangements.

Becker also investigated the negotiation between marriage partners about how to split their family income, as in a prenuptial agreement. However the young from two birds at a nest have an equal number of genes from both parents because of Mendelian inheritance so they can’t negotiate on how many genes each places in their offspring. But they can agree to exchange parentage as side payments with neighbors so that some eggs in the nest come from the pair tending the nest and other eggs from neighbors. According to social selection, extra-pair parentage reflects a system of side payments that stabilizes the social arrangement of economic monogamy when individuals differ in their capacities to contribute to offspring rearing [3].

Overall, the condition for extra-pair parentage to occur is where a high-productivity individual becomes induced to pair with a lower productivity partner whom it would not otherwise prefer. The high-productivity individual then needs to be compensated for the loss of production it would have attained with its preferred partner. In return, the lower productivity partner must concede some proportion of the nest’s parentage as a side payment. The net result is better for both the high and low productivity members of the pair.

A simple numerical example of how to compute a system of pairings and reproductive side payments appears in Roughgarden ([167] Ch. 10, “Sharing Offspring with Neighbors”, pp 228–232).

### 18. Homosexuality

 Over 300 peer-reviewed reports now document or theorize about homosexuality in birds and mammals in a growing literature [1, 11, 12, 41, 48, 57, 62, 119, 143, 158, 165, 172, 207–210]. In some species, homosexuality is mostly between males, in others, mostly between females, and, in still others, both. In some, homosexuality is relatively uncommon, occurring in about 10% of the matings, and in others as common as heterosexual matings, accounting for 50% of all matings.

According to sexual selection, homosexuality is a mistake, deceit or domination. An example deceit theory postulates a small male who sneaks into the territory of a large male, allows the large male to tire by acquiescing to a homosexual copulation, and then proceeds to mate with the females in the large male’s harem. Sexual-selection credits homosexuality as adaptive to only one of the participants—the “gay” animal exploits the “straight” animal or the “straight” animal dominates the subordinate “gay” animal. Other sexual-selection theories portray homosexuality as a genetic defect, maladaptive disease, or a disease caused by genes that decrease fitness in one sex while increasing fitness in the other sex, called sexually antagonistic pleiotropy.

According to social selection, homosexuality is natural, adaptive, and beneficial to both parties [165]. Homosexuality is grouped with other affiliative social behaviors such as mutual grooming, mutual preening, sleeping together, rubbing tongues together, making interlocking calls as well as excess heterosexual mating—all traits that involve the mutual exchange of pleasure. Theoretical interest lies with mutually affiliative behaviors as a class, not solely same-sex mating.

Affiliative behaviors bind animals into a team, allowing them to coordinate actions while providing a sense of one another’s welfare. Teamwork depends on pursuing a common goal as well as on coordinated activity. In social selection, the outcomes of cooperative game theory are realized through team play while pursuing team welfare [173]. Moreover, the act of cooperation itself has evolved to be pleasurable as exemplified by the joy of throwing a touchdown or sinking a basket in human team sports. Teamwork is what makes scoring fun. In basketball, the Alley-Oop pass leading to a dunk is more fun than sinking a foul shot and is followed by high-fives all around.

In humans, the sexual minorities are too common to be a genetic disease according to population-genetic criteria ([165], Ch 16, “Disease versus Diversity”). The fertility cost of male homosexuality can be as much as 50% in contemporary Western culture but much less to nonexistent in other cultures, a cost presumably outweighed by survival advantages leading to a net positive fitness [172]. In humans, the ratio of homosexual to heterosexual individuals may represent a protected polymorphism maintained through frequency-dependent selection between those with bonds maintained through the exchange of power *vs* those with bonds maintained through the exchange of pleasure ([165], Ch 14, “Sexual Orientation”).

Because same-sex matings can be as common as between-sex matings, the geometry of the genitals may have evolved to promote same-sex contact as well as between-sex contact. In bonobos, females participate in same-sex sexuality by facing each other and rubbing their genitals side to side. The position of the clitoris in bonobos is in the front, as in humans, unlike in pigs and sheep, which have clitorises inside their vaginas. Accordingly, de Waal [48] wrote, “The frontal orientation of the bonobo vulva and clitoris strongly suggests that the female genitalia are adapted for this [frontal] position.” More explicitly, Zuk ([226], p 143) wrote that the bonobo clitoris is “frontally placed, perhaps because selection favored a position maximizing stimulation during the genital-genital rubbing common among females.” The frontal clitoral configuration is termed here the “mark of Sappho.” Between-sex mating in bonobos is face to face rather than face to back mounting. The frontal position for heterosexual mating may be how bonobo males adjust to the position of the female genitals, a genital geometry that must work for both same-sex and between-sex sexuality. Thus, genital design enables both sperm transfer as well as social functions.

### 19. Gender multiplicity

 Many species have multiple templates of males and females. Each template is termed a “gender” [165].

Many species of fish, lizards, and birds have a genotype of male whose body-size is large at reproductive age. Such a male must survive several years to attain that size while suffering a high cumulative risk of mortality. But once large, the male can command a territory and defend eggs laid in it. Other males with a different genotype are smaller, reach reproductive age sooner, do not defend territories, and fertilize eggs that are in the territories defended by large males. The two males compete with each other–the large male chases the small one away when it attempts to fertilize eggs in the territory it is defending [13, 66, 88–90, 107, 114, 211].

A three-male pattern is also observed in some fish and birds, where the large male solicits the help of a medium-sized male. The pair of them together maintain the territory and participate in courtship. The large male allows the medium male to fertilize some of the eggs in the territory. The small male meanwhile remains a competitor and fertilizes some of the eggs in spite of the large and medium males’ attempts to chase it away [7, 10, 46, 51, 52, 66, 67, 82, 83, 92, 97, 109, 110, 131, 139, 195, 203–206, 220].

Furthermore, two or more types of females may also occur, laying different sizes of eggs and/or differing in aggressiveness [47, 87, 104, 115, 197].

Species with multiple genders defy sexual-selection theory’s supposition of only one gender per sex. Therefore, sexual selection is augmented with additional narratives.

According to sexual selection, the large territory-holding male is taken as the reference male, and the other types of males are considered as “alternative mating strategies” and defined as “sexual parasites.” The small non-territory-holding male is termed a “sneaker” who “steals” copulations that rightfully belong to the territory-holding male.

Calling the non-reference males pejorative names fosters a theoretically biased research. The word, “sneaker,” when uttered by a teacher or researcher, is accompanied by snickering and guffaws from students and audience. This research culture camouflages locker-room banter as science.

In contrast, social selection defines a “reproductive social group” that extends economic theory for an elemental male-female economic team to a larger team with more “social niches.” A reproductive social group subsumes the concept of a “family”–which is a reproductive social group whose members happen to be genetically related. In a reproductive social group, some members are “pre-zygotic helpers”–animals that assist in bringing about courtship and mating, together with “post-zygotic helpers”–members who remain at the nest to help rear the offspring that have already been born. Those not included in the reproductive social group’s coalition form other arrangements to oppose it, either singly or in coalitions of their own. In this conceptualization, coalitions may form containing medium-sized males who assist in recruiting females to the nests of large males who are guarding the eggs. A large-male-medium-male-female coalition may then be opposed by a small-male coalition that competes to fertilize the eggs.

Discrete multiple genders such as those in fish, reptiles and birds have not been reported in mammals where presumably gender role and expression is more plastic.

So, according to social selection, sex is gametes, all the rest is gender. A body is classified as male or female depending on gamete size whereas the morphology and behavior of that body is gender—a composite trait constructed evolutionarily, developmentally and socially to further the delivery of offspring to the next generation.

As to gender identity in humans, neuro- and developmental biology studies show that the brains of transgender people resemble the brains of the gender they identify with and not with the brains corresponding to their gametic sex. The brains of transgender people develop reflecting the timing of sex hormones at different stages during fetal growth (genitals early, brain late, Savic, *et al.* [182], Swaab *et al.* [194]). Gender identity has developed by the time of birth, and sexual orientation may develop later during the first few years of infancy. Gender identity may exist in the brain as a cognitive lens or receptive field that controls who to focus on as a developmental ‘tutor.’ Transgender identity is the acceptance of a tutor from the opposite sex (*cf.* [172]).

The acquisition of gender identity could be studied in duetting songbirds such as the canebrake wren [159]. Chicks in these and other duetting species must somehow determine which parent has a sex corresponding with their own, and then learn their song from that parent. Undoubtedly a few chicks learn the song of the parent not corresponding to their own sex, leading to transgender birds.

### 20. Feminine males

 In species with three male genders, one gender may have colors or markings shared with females. Sexual selection describes these feminine males as mimics who steal the reproductive investment of territory-holding males through deceit. The female mimic fools the territory-holding male to allow its entry into the harem whereupon it mates with the harem females.

The concept of mimicry is borrowed from predator-prey interactions. A Batesian mimic is a fly that resembles a bumble bee to avoid predation. The resemblance is exact—one must look closely through a magnifying glass to distinguish a Bayesian-mimic fly from a bee. A bird on the wing has a split second to decide whether to grab an insect and doesn’t have the time to investigate the bug’s real identity.

Unlike true mimicry, the resemblance of a feminine male to a female is far less than perfect and the male has a long time for inspection. In the blue-gill sunfish, the masculine territory-holding male solicits the feminine male to join him through a courtship ritual. Masculine males behave differently to feminine males than to females, showing they are aware of the distinction. Deceit narratives require great asymmetry in gullibility. The masculine male must be too dumb to know the difference between a male and a female, while the feminine male must be smart enough to deceive the masculine male. Yet fish, lizards, and birds are visual, diurnal animals who make a living with their eyesight and draw fine distinctions in their choice of prey. The concept of a female mimic is another locker-room fantasy of sexual-selection theorists—a straight guy fooled by a drag queen at a bar. Some sexual-selection workers borrow the phrase “she-male” from pornography to describe male-male courtship in garter snakes [120, 188], 2003b instead of using the recommended term, “gynomorphic male” [79].

According to social selection, markings and colors on animals represent “body English”–how animals tell one another what their social role is, what their intentions are, and what activities they promise to perform. The markings and colors indicate an occupation, like a work uniform does in humans. Feminine males participate in conversations with words used more frequently by females than by the territory-holding males. Through courtship, a territory-holding male Ruff hires a feminine male who flocked or schooled with females while he was setting up his territory. The feminine male is a “marriage broker” who assists in recruiting females to lay eggs in the territory. With his body English, he can attract and communicate with females reassuring them that the territory-holding male will not abandon the eggs [165].

### 21. Masculine females

 In sexual-selection theory, masculine females are discussed under the rubric of “female ornaments”–hanging skin flaps (wattles), colored patches of feathers, antlers, and so forth [8, 9, 40, 45, 53, 156, 221–224]. Among insects, damselflies have masculine females [58]. Hummingbirds have both feminine males and masculine females [18, 19]. Darwin dismissed out-of-place ornaments as male traits accidentally expressed in females, a developmental error.

According to social selection, masculine females are the reverse of feminine males, namely, a female using body English to converse on topics and with words used more frequently by males than by the feminine females. Such conversations might involve establishing and defending territories in species where this task is might otherwise be carried out by males. Masculine females appear underreported because feminine males draw more sensational attention.

In social selection, ornaments, both male and female, serve as “admission tickets” to power-holding cliques that control the opportunity for successful rearing of offspring. Admission tickets are expensive because the advantage to membership in a clique resides in the power of monopoly, which is diluted when membership is expanded. By requiring a high price of admission, the monopolistic coalition is kept exclusive, maximizing benefit to those within. Ornamental admission tickets belong to a class of traits called “social-inclusionary traits” that are needed to participate in the social infrastructure within which offspring are reared. Not possessing such traits, or not participating in social-inclusionary behaviors, is reproductively lethal.

### 22. Sexual monomorphism

 In sexual selection, sexual dimorphism is presupposed. Yet in many species, males and females are virtually indentical, where sexes can only be distinguished by careful inspection of the genitals. Sexual-selection theory is silent on why species vary from the highly sexually dimorphic peacock and to the sexually monomorphic penguin. Sexual monomorphism is problematic for sexual selection. One workaround posits a strategic concealment of sexual identity [108].

In social-selection theory, sexual monomorphism represents males and females both employing the same body English because the negotiated division of labor involves both sexes doing the same tasks.

### 23. Human attractiveness

 Sexual-selection theory is axiomatic in evolutionary psychology. Evolutionary psychologists uncritically extrapolate sexual-selection narratives to humans. They claim to explain what males regard as beauty and claim women find men handsome who reveal their genetic quality [25].

According to social selection, what males and females find attractive correlates with a potential to successfully deliver offspring to the next generation.

### 24. Human rape

 Sexual selection views human rape as an evolutionary strategy whereby men who are not preferred as mates by women manage to reproduce through coercion [198].

Social selection views human rape as domination, an exercise of power that can be either homosexual or heterosexual, and that has little relation to reproduction.

### 25. Human brain

 Sexual-selection theorists posit the human brain is an ornament used by men to attract women [130]. One imagines a man using his ornamental brain to compose lovely sonnets to woo a mate. The problem then is to explain why women have brains, inasmuch as only males possess ornaments according to sexual selection. So, sexual selection theorists postulate that females have brains to appreciate the brains of men–only big-brained women can appreciate the sonnets of big-brained men.

Social selection views the human brain a trait is equally necessary in both men and women to successfully deliver offspring to the next generation.

### 26. The selfish world

The preceding paragraphs presented a broad sweep of issues pertaining to sexual reproduction in which the sexual-selection explanation was either falsified by data or for which supportive data are lacking. Could the lack of explanatory success of sexual selection on so many topics be an unfortunate coincidence? Suppose the chance of sexual selection being right on any one topic is 1/2 and being wrong is also 1/2. Per the preceding paragraphs, sexual selection is apparently wrong on 25 topics. Then the odds of being incorrect on all 25 topics assuming independence is $(1/2)^{25}$, which equals about $3 \times 10^{-8}$. This low probability implies that the assumption of independence must be rejected. Instead, the sexual-selection positions on all 25 topics are coupled. Although one may tinker with this calculation, the point is that some feature common to all 25 topics in sexual selection must explain why they are all incorrect at the same time. That feature is that all sexual-selection positions derive from a common view of animal behavior predicated on selfishness, deception, and genetic weeding. And if the sexual-selection view of biological nature is indeed wrong, then developing additional sexual-selection positions on other topics will fail as well. For this reason, the social-selection stance is that the sexual-system system cannot be repaired or sanitized. Its foundation is incorrect to begin with.

As previously stated, the issue is not whether a biological nature predicated on selfishness, deception, and genetic hierarchy is appealing or repugnant compared with a biological nature predicated on teamwork, honesty, and genetic equality. The issue is which of these views of biological nature is true. The preceding paragraphs collectively demonstrate that the selfish-gene picture of nature is not accurate.

Some feel that the selfish gene metaphor merely expresses a darkly poetic vision of natural dystopia, an entertaining hyperbole, but otherwise inconsequential. However, the preceding paragraphs show that the selfish gene metaphor is more than mere poetry. It underwrites an extensive scientific theory that purports to explain some of the basic questions of life that humankind has ever posed. And, because that metaphor is inaccurate, a huge body of scientific theory is incorrect. In contrast, the social-selection system addresses the same basic questions of life that sexual-selection system does, but does so with a picture of nature that presently appears true and accurate. Time will tell whether social selection is indeed correct or whether some substantial modification or third approach is needed.

## Responses

This article presents two alternative sex-gender systems–sexual selection that emphasizes mating and social selection that emphasizes offspring delivery. The top three fatal problems with sexual selection are: (1) the paradox of the lek that rules out a hierarchy of genetic quality in males, (2) sex role reversal that rules out a link between the sperm/egg size difference and sex roles, and (3) its inadequacy to address sexual diversity beyond a heterosexual binary, necessitating implausible workarounds. Because of these fatal problems, the social selection perspective is that sexual selection cannot be fixed, and should be replaced. What then have been responses to this broad challenge to the sexual selection system?

Some don’t see a problem. “Darwin’s theory of sexual selection was one of his most brilliant accomplishments and perhaps the one that has been the least well understood...The Descent of Man, and Selection in Relation to Sex deserves to be read as a profound contribution to the philosophy of our subject” [64]. Similarly, “Sexual selection has come to be seen as a keystone of Charles Darwin’s theory of evolution by natural selection” [112].

Others do see a problem and propose amendments. Clutton-Brock [32] concedes: (1) “Sex differences in parental care are not an inevitable consequence of sex differences in gamete size because patterns of parental care are likely to co-evolve and feedbacks may be complex”. (2) “in a number of birds where females and males have similar ornaments, both sexes are commonly involved in aggressive displays with rivals”.

Therefore, Clutton-Brock [32] develops a mirror-image narrative for female-female competition and mate choice by males for the best females. He writes that, “intra-sexual competition between females for resources may generate large individual differences in fecundity that strengthen selection on males to identify and prefer superior partners and selection on females to signal temporal and individual differences in fecundity.” To expand sexual-selection by developing a mirror image narrative for female-female competition with male choice of the best female, occurring simultaneously with male-male competition and female choice of the best male, fails for the same reasons already raised. Doing a global search and replace transposing “male” and “female” produces difficulties appropriate to a female-oriented mirror-image narrative. For example, if males are choosing the females genetically best at raising young, then soon the genetic variation for female quality disappears, yielding what is termed here the “paradox of the creche,” which is the mirror image of the paradox of the lek.

Nonetheless, Clutton-Brock [32] concludes that “the theory of sexual selection still provides a robust framework that explains much of the variation in the development of secondary sexual characters in males, although the mechanisms controlling the relative intensity of reproductive competition and the relative development of secondary sexual characters in the two sexes are more complex than was originally supposed. The debate about the viability of sexual selection as a framework continued as a back-and-forth in Roughgarden and Akçay (2010ab), Clutton-Brock [33], Shuker [190] and Roughgarden [171].

The feminist critique of sexual selection also leaves the skeleton intact. Hrdy [91] writes, “competition between those of one sex for reproductive access to the other remains a robust explanatory framework”. West-Eberhard [219] extends sexual-selection reasoning to “resources” other than mates (1983). Zuk opposes the need to replace sexual selection [99]. And after demolishing Bateman’s [14] experiments, Snyder and Gowaty [193] write: “We do not intend this reanalysis as a criticism of Bateman...We easily attributed two of the mistakes to rounding errors, but the other appears to be an error in arithmetic—acceptable in an era before calculators.”

Hoquet’s [84, 85] review of the difficulties with Bateman’s experiments led Morimoto [128] to dismiss Bateman’s mistakes, writing, “it is almost irrelevant that some studies might have failed to replicate Bateman’s original experimental design to its finest details” and that “Bateman’s principles have undergone significant changes since their original conceptualization”. Yet he still concludes that “the fact that Bateman’s principles provide a valuable quantitative framework in the field of sexual selection is unquestionable.”

Thus, the responses by sexual-selection researchers argue that many of the criticisms of sexual selection are valid but that the sexual selection framework has been patched and remains suitable for future work.

A workshop held in 2013 to assess the state of sexual selection studies revealed that sexual-selection researchers could not agree on the definition of sexual selection. Therefore, a subcommittee of participants developed the “NESC2015 definition”, named for the workshop’s meeting site at the National Evolutionary Synthesis Center in North Carolina. The definition contains six elements [177]: (1) Sexual selection is a differential probability of the genotypes within a sex being incorporated into fertilizations independent of a difference in total fecundity; (2) The definition does not specify paradigmatic sex roles for males and females; (3) Sexual selection consists of processes that increase share of gene pool; (4) Fecundity selection consists of processes that increase size of gene pool; (5) Mating behavior is social and/or reproductive; (6) Selection favors achieving offspring delivery via negotiated and coordinated cooperation.

The NESC2015 definition is deliberately neutral with respect to male and female sex roles. It also distinguishes a process of sexual selection from fecundity selection. It further stipulates that mating behavior serves both social and sperm transfer purposes, that selection favors offspring delivery rather than fertilizations *per se* and that successful offspring delivery is achieved through negotiated and coordinated cooperation.

The NESC2015 report explains the distinction between sexual selection and fecundity selection in this way: If a female chooses one male over another solely because of his color, without any impact on egg production, then sexual selection occurs. Alternatively, if the female chooses one male over another because of the quantity of resources he supplies, then both fertility selection and sexual selection occur together. Both male and female prosper from the increased egg production and additionally the male prospers because of being selected over males supplying fewer resources. The report includes an example of how to model negotiation in the behavioral tier of bi-parental care leading to a Nash bargaining solution that maximizes clutch size by including an optimal amount of both sexual and fecundity selection.

Shuker and Kvarnemo [191] also reviewed the definition of sexual selection. They write “Our main aim in writing this paper is to allow the next generation of sexual selection researchers to address the many questions still left unanswered without the baggage of the definition of sexual selection left lying around.” They confirm the NESCent finding that no generally accepted definition of sexual selection exists. Table 2 in their article presents a full page typeset in fine print showing the many definitions of sexual selection presently in use. Shuker and Kvarnemo [191] also present their own definition, denoted as SK2021: (1) Sexual selection is any selection that arises from fitness differences associated with nonrandom success in the competition for access to gametes for fertilization; (2) Definition is explicitly sex and sex-role neutral; (3) In sexual selection, gametes are the target of selection; (4) In natural selection, resources are the target of selection; (5) Mating behavior is for access to gametes; (6) Selection favors winning at competition for mates.

NESC2015 and SK2021 agree that the longstanding association of sexual selection with sex roles including a relation of gamete size to sex role is discredited. Both NESC2015 and SK2021 are sex neutral. NESC2015 distinguishes between sexual and fecundity selection whereas SK2021 distinguishes between sexual and natural selection. The intent, though not the wording, seems similar in both distinctions.

However, NESC2015 and SK2021 do have significant points of disagreement. First, in NESC2015 mating serves *both* social and sperm-transfer purposes, whereas in SK2021 mating is solely for access to gametes. Second, in NESC2015 selection is about increasing offspring delivery via negotiated and coordinated cooperation whereas in SK2021 selection is about winning at competition for mates. By both definitions selection results in an increase of genes in the gene pool, but the definitions differ on the behavioral mechanisms used to attain that increase.

From a social-selection perspective it’s a mistake for SK2021 to stipulate the word, competition, in the definition. The definition should be neutral with respect to competition and cooperation. To stipulate competition invites two rhetorical traps. First is the need to view cooperation as competition in disguise, for example, by claiming that two males are “really” just competing when offering different degrees of cooperation to a female—such phrasing privileges competition over cooperation. Second is the need to specify an object of competition which SK2021 do with a distinction between gametes and resources as the objects of competition. Courtship behavior is not about competition for objects, but is a system for exchanging bids in the currency of fitness.

The deepest analysis of social selection to date occurred during a session at the International Society for the History, Philosophy and Social Studies of Biology (ISHPSSB) held in 2009 in Brisbane, Australia that featured a perspective from the history and philosophy of science [124].

Milam [124] writes that, “I worry that her attempts to dismiss sexual selection as invalid have obscured the potential implications of her theory of social selection”. Milam calls attention to antecedents from the early to mid 1900s to ideas in social selection theory and concludes, “Roughgarden’s theory of social selection is not really an alternative to sexual selection as much as it is a return to a set of possibilities and convictions that dominated biologists’ discussions of the evolution of social behavior and female choice before the rise of sociobiology in the 1970s. Yet by framing social selection as an alternative to sexual selection, Roughgarden sells herself short. Discussions surrounding the *Genial Gene* do not have to be only arguments over the validity of sexual selection; they could instead be productive discussions about the evolutionary basis of sociality, based on what we have learned about the evolution of animal minds and culture in the 100 years since Morgan.”

Millstein [124] offers a table clarifying how to distinguish natural selection from sexual selection from social selection. She concludes “In short, Roughgarden is vindicated in offering a revolutionary alternative to sexual selection and not a mere revision to existing views. And Darwin is vindicated in his separation of natural selection from sexual selection.”

Potochnik [124] writes “Roughgarden’s claim that she has falsified the view that selfishness is basic is incorrect....Roughgarden should not aim to falsify a commitment to selfishness but to demonstrate its unfruitfulness...If Roughgarden is right that flawed evolutionary explanations result from the assumption of widespread selfishness, perhaps the proper lesson is not to assume widespread cooperation, but to avoid committing to any view of what is basic to biological nature...Perhaps the true value of the claim that cooperation is basic is in its power to undermine implicit assumptions of the opposite.”

Finally, an anonymous reviewer of this manuscript observed that social selection makes “an appeal to economic game theory and similar tools...At times it seems like the author is offering an evil twin of sexual selection theory rather than a radically different way of approaching this science. Part of the paradigm shift that will finally end the domination of sexual selection theory will include a deep analysis of the ways in which our values impinge on the scientific process, including which tools we use. Any application of economic theory to social relations comes laden with assumptions and value judgments on how behaviour arises, the motivations behind behaviour, and its possible payoffs. Without pumping the brakes and deeply critiquing the earliest stages of the inquiry process, I fear social selection theory falls victim to similar problems as sexual selection theory.”

Guilty as charged. Social selection theory lies entirely within evolutionary theory including its methods—its premise is that sexual selection theory has incorrectly carried out the evolutionary explanation of sexual reproduction and associated traits. Social selection theory does not challenge evolutionary theory writ large. That awaits a future critique should both social selection and sexual selection theories ultimately prove incorrect.

## Conclusion

The sexual-selection system features bad genes, selfishness, competition, conflict, coercion, ownership and deceit. It is misogynistic. It is pejorative to gender and sexual minorities. It is genetic classism. It promotes genetic entitlement. It naturalizes a genetic royalty endowed with good genes. It is a political project.

The social-selection system features negotiation, teamwork, division of labor and genetic equality.

If the sexual-selection system is true, so be it. But if false, scholars should acknowledge its death lest it linger as an excuse for injustice.

## Data Availability

No datasets were generated or analysed during the current study.

## Footnotes

1 The geometric mean through time of each generation’s arithmetic mean fitness based on the current Hardy-Weingberg frequencies is higher in a random-mating diploid sexual population than in a corresponding asexual population. Long-term survival of a population depends on a high geometric mean fitness.

2 The term hermaphrodite is used in biology to denote an organism that makes both eggs and sperm at some time during its life. In medicine the term hermaphrodite, synonymous with intersex, denotes an organism whose embryonic development has produced both male and female morphological traits in the same individual. In medicine the term hermaphrodite has a negative or anomalous connotation.

3 Dioecy is often used with plants and gonochorism with animals, but their meaning is synonymous. In this article, dioecy is used throughout.
